# Ca^2+^ is a key factor in α-synuclein-induced neurotoxicity

**DOI:** 10.1242/jcs.180737

**Published:** 2016-05-01

**Authors:** Plamena R. Angelova, Marthe H. R. Ludtmann, Mathew H. Horrocks, Alexander Negoda, Nunilo Cremades, David Klenerman, Christopher M. Dobson, Nicholas W. Wood, Evgeny V. Pavlov, Sonia Gandhi, Andrey Y. Abramov

**Affiliations:** 1 UCL Institute of Neurology, Queen Square, London WC1N 3BG, UK; 2 Department of Chemistry, University of Cambridge, Cambridge CB2 1EW, UK; 3 Department of Physiology and Biophysics, Dalhousie University, Halifax B3H 4R2, Canada; 4 College of Dentistry, New York University, New York 10010, USA

**Keywords:** α-Synuclein, Ca^2+^ signalling, Parkinson's disease, Neuronal death

## Abstract

Aggregation of α-synuclein leads to the formation of oligomeric intermediates that can interact with membranes to form pores. However, it is unknown how this leads to cell toxicity in Parkinson's disease. We investigated the species-specific effects of α-synuclein on Ca^2+^ signalling in primary neurons and astrocytes using live neuronal imaging and electrophysiology on artificial membranes. We demonstrate that α-synuclein induces an increase in basal intracellular Ca^2+^ in its unfolded monomeric state as well as in its oligomeric state. Electrophysiology of artificial membranes demonstrated that α-synuclein monomers induce irregular ionic currents, whereas α-synuclein oligomers induce rare discrete channel formation events. Despite the ability of monomeric α-synuclein to affect Ca^2+^ signalling, it is only the oligomeric form of α-synuclein that induces cell death. Oligomer-induced cell death was abolished by the exclusion of extracellular Ca^2+^, which prevented the α-synuclein-induced Ca^2+^ dysregulation. The findings of this study confirm that α-synuclein interacts with membranes to affect Ca^2+^ signalling in a structure-specific manner and the oligomeric β-sheet-rich α-synuclein species ultimately leads to Ca^2+^ dysregulation and Ca^2+^-dependent cell death.

## Abstract (precis)

**Summary:** Monomeric and oligomeric α-synuclein induce Ca^2+^ signal in neurons and astrocytes by incorporating into the membrane.

## INTRODUCTION

Neurodegenerative diseases share a common pathological process of misfolding of a native monomeric protein into a range of intermediate oligomeric structures, and finally polymerising into insoluble amyloid fibrils and depositing in the brain as inclusions. In Parkinson's disease, it is the native protein α-synuclein that undergoes self-aggregation and deposition in Lewy bodies (Cookson, 2009). Extensive evidence now suggests that the smaller and soluble intermediate products of α-synuclein aggregation (termed oligomers) are likely to be the pathogenic culprits of disease (Kalia et al., 2013). During the aggregation process, monomeric α-synuclein initially forms oligomers (2mer–50mers) that are unstructured, degradable and non-toxic to cells. These then undergo a conversion into highly compact and β-sheet structured oligomers that are stable and toxic to cells (Cremades et al., 2012). Complementary *in vivo* studies have demonstrated that overexpression of oligomer-forming variants of α-synuclein in rat substantia nigra induces significant dopaminergic neuronal death, whereas overexpression of fibril-forming variants of α-synuclein does not demonstrate significant toxicity (Winner et al., 2011). Intermediate species of aggregated α-synuclein might also be transferred between neurons and induce toxicity as well as seed further aggregation in recipient neurons *in vitro* (Desplats et al., 2009) and *in vivo* (Hansen et al., 2011; Volpicelli-Daley et al., 2011). However, the nature and mode of action of the structural forms of these toxic intermediate aggregates remains unclear.

Ca^2+^ dysregulation has been reported previously in α-synucleinopathy models of Parkinson's disease, and there are a number of ways in which α-synuclein and Ca^2+^ might be linked. Overexpression of intracellular α-synuclein in neuroblastoma cell models has been associated with alterations in basal and depolarising-stimulus-evoked Ca^2+^ signals (Danzer et al., 2007). α-Synuclein itself, in its annular oligomeric form, can induce a Ca^2+^ flux across artificial membranes and neuronal membranes through pore-forming mechanisms (Danzer et al., 2007). High levels of intracellular Ca^2+^ induced either by thapsigargin, ionophores or depolarising stimuli can promote the intracellular oligomerisation and aggregation of α-synuclein. The removal of Ca^2+^ in these experiments is able to prevent α-synuclein aggregation. Thus, there is a complex loop in which α-synuclein expression and abnormal aggregation might initially promote Ca^2+^ dysregulation, which might in turn promote further aggregation (Follett et al., 2013; Nath et al., 2011).

Although it has been reported that oligomers of aggregated proteins might induce Ca^2+^ fluxes across membranes *in vitro*, the exact structural species of the protein responsible, the source of the Ca^2+^ signal in cells, the impact on physiological Ca^2+^ signalling and the role in neuronal death have not been elucidated. In this study, we have utilised a range of biophysical methods, live neuronal imaging and electrophysiology to dissect which conformational variant of α-synuclein induces a Ca^2+^ signal in neurons, the mechanism of α-synuclein-induced Ca^2+^ dysregulation and the consequence of Ca^2+^ dysregulation on cell toxicity.

## RESULTS

### Monomeric and oligomeric α-synuclein both induce a [Ca^2+^]_c_ rise in neurons and astrocytes

**Fig. 1.**
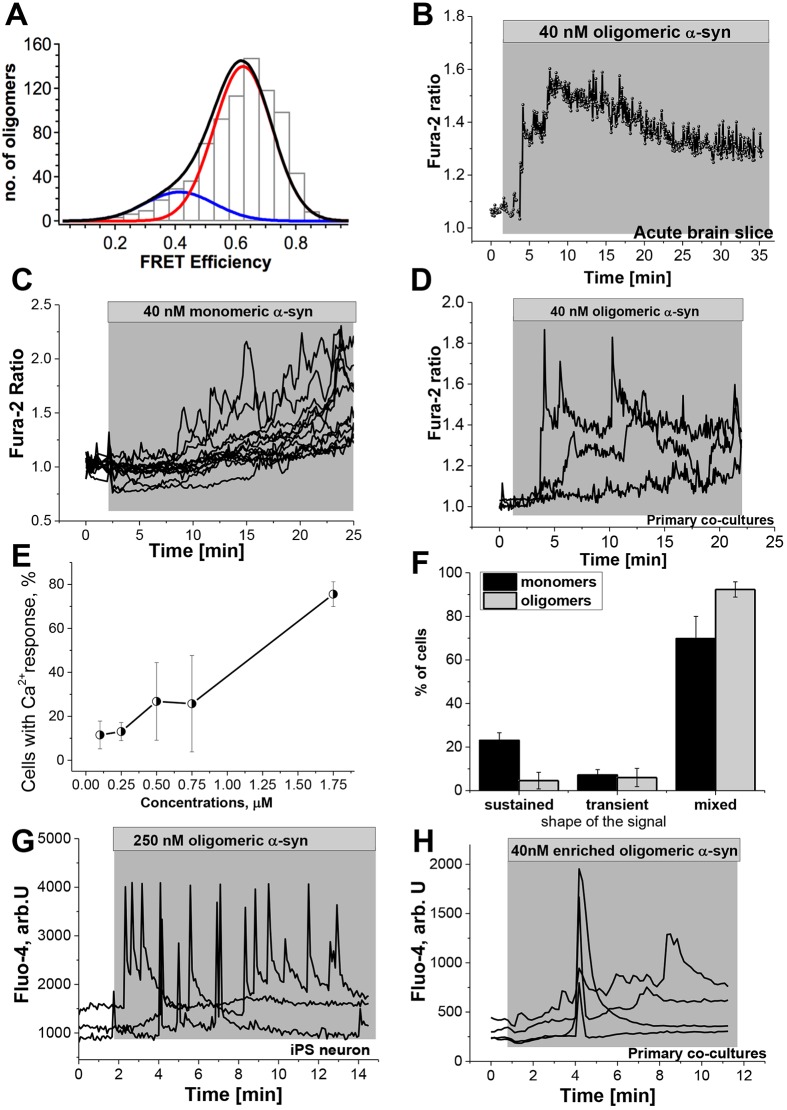
**Application of extracellular α-synuclein induces a cytosolic Ca^2+^ signal.** (A) A characteristic single-molecule FRET histogram of the 29-h time-point oligomerisation was used in this study. There are both globular non-toxic (18%, E=0.41, blue line), and toxic β-sheet-containing oligomers (82%, E=0.62), red and black lines, present. (B) Application of 40 nM oligomeric α-synuclein (α-syn) induces a Ca^2+^ signal in an acute rat brain slice. (C,D) 40 nM of monomeric (C) or oligomeric (D) α-synuclein induced an elevation of cytosolic Ca^2+^ signal in primary neurons and astrocytes. Both forms of α-synuclein induced a rise in basal cytosolic Ca^2+^ as well as Ca^2+^ spikes. (E) Dose–response experiment demonstrating the number of cells responding at different concentrations of oligomeric α-synuclein. (F) Histogram demonstrating the proportion of cells exhibiting each pattern of Ca^2+^ signal. (G) The α-synuclein-induced Ca^2+^ signal was also observed in neurons derived from iPSCs. (H) Enriched oligomeric α-synuclein induced a rise in basal cytosolic Ca^2+^ as well as Ca^2+^ spikes. Results are mean±s.e.m. [*n*=98 for monomers; *n*=183 for oligomers (E,F)].

We wished to determine whether α-synuclein itself was able to induce a Ca^2+^ signal, and which conformational state determined this signal. We therefore generated monomeric and oligomeric species of α-synuclein and tested their effect on primary neurons and astrocytes, induced pluripotent stem cell (iPSC)-derived human neurons, and acute brain slices. We utilised two different methods for the preparation of different α-synuclein species. In method 1, we add Alexa Fluor 594 (AF594)- or Alexa Fluor 647 (AF647)-labelled monomeric α-synuclein to Alexa Fluor 488 (AF488)-labelled monomeric α-synuclein in a 1:1 ratio. As the aggregation proceeds, oligomers consisting of both the AF488–α-synuclein and the AF594–α-synuclein will form, and, as the AF488–α-synuclein acts as a FRET donor, and the AF594–α-synuclein as a FRET acceptor, we can use single-molecule confocal FRET analysis to differentiate the oligomeric α-synuclein from the rest of the monomeric protein. Moreover, we can further utilize the FRET efficiency analysis to determine the formation of the compact β-sheet-containing oligomers (Cremades et al., 2012). Following aggregation for 29 h, we detect ∼0.8% oligomers (number of detected oligomers as a fraction of total number of detected events). These consist of two populations of oligomers: 82% formed a high FRET efficiency population (E=0.62; Fig. 1A) characteristic of the compact β-sheet oligomers previously identified (Cremades et al., 2012), and 18% had formed a low FRET efficiency oligomer population (E=0.41, Fig. 1A).

As this method generates a mixture of monomers and oligomers, we also utilised a second method based on lyophilisation (method 2) to generate an enriched oligomer preparation, which allowed us to determine oligomer-specific effects on cells. The enriched oligomers generated in this method have been extensively characterised and reported previously (Chen et al., 2015). Using this method, we were able to generate a preparation containing >90% oligomers. These oligomers are of cylindrical structures, composed of 10–40 protein molecules and a β-sheet content of 30-40%.

It is well established that α-synuclein is present extracellularly, and we, and others, have shown that it is rapidly taken up by neurons and astrocytes in its monomeric, oligomeric and fibrillar forms (Cremades et al., 2012). Application of monomeric and oligomeric α-synuclein induces a cytosolic Ca^2+^ signal in both neurons and astrocytes. In these experiments, cells loaded with the Ca^2+^ indicator were initially observed at basal levels for 2 min prior to application of α-synuclein. Cells that exhibited spontaneous oscillations prior to addition of α-synuclein were excluded from the analysis. The α-synuclein species was then added to the cells and recordings are taken for 30–40 min. Two patterns of α-synuclein-induced Ca^2+^ signal were observed. Oligomeric α-synuclein induces a transient Ca^2+^ signal, consisting of Ca^2+^ spikes, with and without complete recovery to baseline, at a concentration range of 40–250 nM, after 2–6 min (Fig. 1D; *n*=189). Monomeric α-synuclein induces transient Ca^2+^ spikes superimposed on a gradual increase in the basal Ca^2+^ signal (Fig. 1C; *n*=568). We report that the amplitude and shape of the α-synuclein-induced Ca^2+^ signal was consistently one of these patterns, and appeared to be independent of the concentration of α-synuclein applied. Fig. 1E demonstrates the number of cells exhibiting a Ca^2+^ response in a dose–response experiment performed on cells of the same batch at the same number of days *in vitro* (DIV), using the same preparation of aggregated α-synuclein. At concentrations of 100 nM (oligomer 1 nM), 11.5±6.3% cells responded; at 250 nM (oligomer 2.5 nM), 13.0±4.1% cells responded; at 500 nM (oligomer 5 nM), 26.8±17.6% cells responded; at 750 nM (oligomer 7.5 nM), 25.7±21.9% cells responded; at 1.75 µM (oligomer 17.5 nM); 75.6±5.6% cells responded (mean±s.e.m.). In summary, the amplitude of the α-synuclein-induced Ca^2+^ signal did not vary with dose. However, the number of cells in the culture that responded to extracellular α-synuclein did vary, increasing with higher concentrations of α-synuclein. Fig. 1F demonstrates the quantification of the observed patterns of Ca^2+^ signalling induced by α-synuclein in the representative dose–response experiment shown in Fig. 1E. Monomers alone induced a sustained rise in basal Ca^2+^ (23.1±3.5%, *n*=32), or transient spikes (7.1±2.5%, *n*=7), or a mixed pattern (69.8±10.2%, *n*=59). Oligomers induced a sustained rise in basal Ca^2+^ (4.6±3.8%, *n*=3), or transient spikes (6.0±4.2%, *n*=11) or a mixed pattern (92.4±3.5%, *n*=169). Thus overall, we show that the majority of cells will exhibit both transient Ca^2+^ spikes as well as a sustained rise in basal Ca^2+^. A small proportion of cells will demonstrate exclusively a sustained rise in basal Ca^2+^ particularly on exposure to monomers.

The physiological concentrations of extracellular α-synuclein are estimated to be in the low nanomolar range for monomer (based on the estimated concentration of extracellular α-synuclein in cerebrospinal fluid studies), and, as only 1% of the monomeric form aggregates, it is estimated to be in the picomolar range for oligomers. We were able to detect α-synuclein-induced Ca^2+^ signals as low as 10 nM (for monomers) and 100 pM (for oligomers). However, at these concentrations <5% of the cells in the culture responded. Therefore, for all further experiments, concentrations of α-synuclein were employed that ensured a Ca^2+^ signal in at least 30% of cells. Of note, the α-synuclein-induced Ca^2+^ signal was independent of cell type and was observed in both neurons and astrocytes, and was also independent of brain region as it was observed in primary co-cultures from midbrain and cortex, but also from human cortical neurons derived from iPSCs (Fig. 1G). Furthermore, the oligomer-induced Ca^2+^ signal was also observed in *ex vivo* brain slices from rat cortex loaded with fura-2 (Fig. 1B, *n*=59 cells from 5 slices).

As both the monomeric and oligomeric α-synuclein induced a Ca^2+^ signal in neurons, we also applied the enriched oligomer preparation (from method 2) to primary co-cultures to determine whether oligomeric α-synuclein alone induced a Ca^2+^ signal. We observed a similar α-synuclein Ca^2+^ signal (*n*=5 experiments; Fig. 1H) to that observed with the oligomeric α-synuclein generated by method 1.

### The α-synuclein-induced Ca^2+^ signal is dependent on influx of extracellular Ca^2+^

**Fig. 2.**
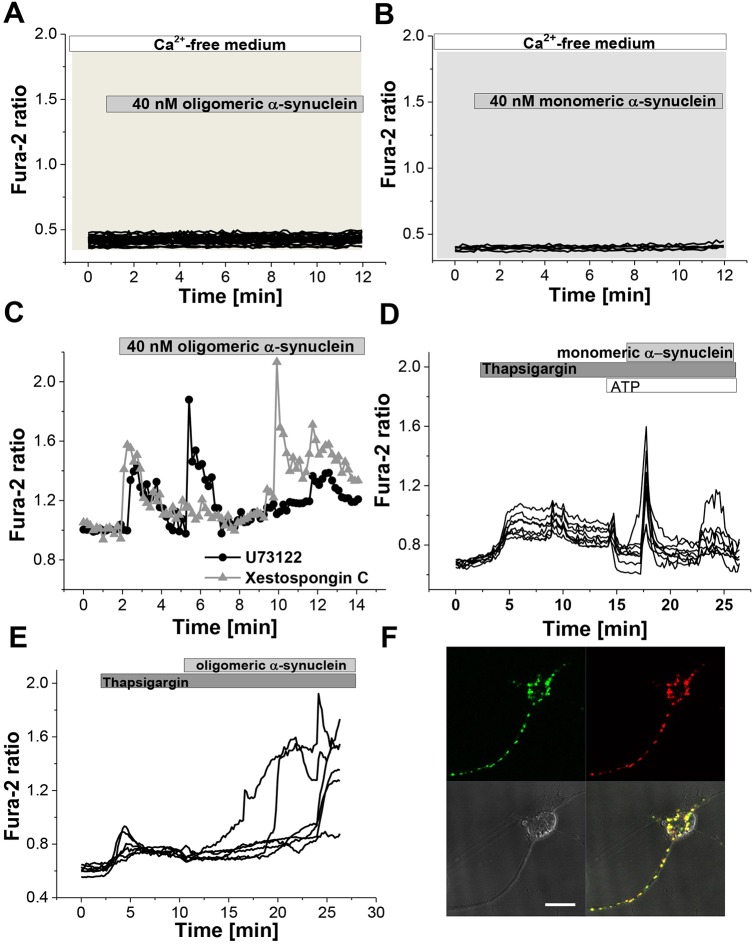
**Identification of the source of the α-synuclein-induced Ca^2+^ signal.** (A,B) Both monomeric and oligomeric Ca^2+^ signals could be prevented completely by removal of Ca^2+^ from the extracellular medium. (C) Inhibition of PLC by U73122 and inhibition of IP^3^ receptors by Xestospongin C had no effect on the α-synuclein-induced Ca^2+^ signal. (D,E) Depletion of the ER Ca^2+^ store using thapsigargin did not affect the monomeric or oligomeric-induced Ca^2+^ signal. (F) Representative image of single neuron following application of equimolar ratio of α-synuclein labelled with monomeric AF488 (green) and AF647 (red) demonstrating intracellular uptake of recombinant α-synuclein species. Scale bar: 10 μm.

Next, we investigated the source of the α-synuclein-induced Ca^2+^ signal in primary co-cultures. We applied monomeric and oligomeric α-synuclein to wild-type primary cells in Ca^2+^-free medium (Ca^2+^-free HBSS, 0.5 mM EGTA and 1.25 mM MgCl_2_) and demonstrated that α-synuclein no longer induced any Ca^2+^ signal (Fig. 2A,B), suggesting that external Ca^2+^ is crucial for the α-synuclein-induced Ca^2+^ signal.

The transient and oscillatory [Ca^2+^]_c_ signals mirror those seen in endoplasmic reticulum (ER) [Ca^2+^]_c_ release induced by inositol 1,4,5-trisphosphate (IP_3_). External Ca^2+^ can activate phospholipase C (PLC), which could in turn generate IP_3_ and thus mobilise ER Ca^2+^. To assess this hypothesis, we employed U73122 (5 µM), an inhibitor of phospholipase C (Fig. 2C), which did not significantly alter the monomeric- or oligomeric-α-synuclein-induced [Ca^2+^]_c_ astrocyte and neuronal signals (*n*=98 cells). Similarly, Xestospongin C (10 µM), an inhibitor of IP_3_-dependent Ca^2+^ release, failed to reduce the oligomeric-α-synuclein-induced [Ca^2+^]_c_ signals in astrocytes and neurons (*n*=123 cells; Fig. 2C). Both U73122 and Xestospongin C completely blocked the ATP-induced Ca^2+^ signal in astrocytes at these concentrations (Domijan et al., 2014). Therefore, these data confirm that PLC- and IP_3_-mediated Ca^2+^ signalling does not contribute significantly to the α-synuclein-induced [Ca^2+^]_c_ responses.

We applied 0.5 µM thapsigargin (an inhibitor of ER Ca^2+^ pumps) to primary co-cultures in order to deplete Ca^2+^ from the ER. To ensure that the concentration of thapsigargin was adequate for complete ER depletion of Ca^2+^, ATP was then applied to the culture, and this did not induce a cytosolic Ca^2+^ response. This was followed by the addition of monomeric (Fig. 2D; *n*=199) or oligomeric (Fig. 2E; *n*=218) α-synuclein, which still induced a similar [Ca^2+^]_c_ response as before. Taken together, these data strongly suggest that ER Ca^2+^ does not contribute to the α-synuclein-induced [Ca^2+^]_c_ responses.

**Fig. 3.**
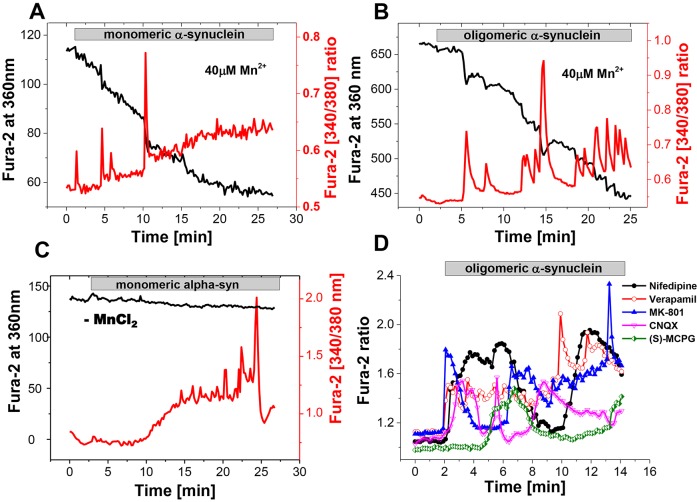
**Mn^2+^ quench assay confirms that α-synuclein-induced Ca^2+^ transients originate from a Ca^2+^ influx across plasmalemmal membrane.** (A,B) Fura-2-loaded neurons showed typical [Ca^2+^]_c_ fluctuations in response to monomeric (A) or oligomeric (B) α-synuclein. With the addition of 40 µM Mn^2+^, each [Ca^2+^]_c_ transient was accompanied by a step quench of the 360-nm fura-2 signal, confirming that each transient reflects a pulsed influx of divalent cations seen in response to α-synuclein. (C) Application of monomeric α-synuclein in the absence of MnCl_2_ did not induce any alteration in the fura-2 360-nm signal. (D) The α-synuclein-induced Ca^2+^ influx was independent of the presence of inhibitors of plasmalemmal channels [20 µM verapamil, 1 µM Nifedipine, 10 µM MK-801, 20 µM CNQX or 50 µM (S)-MCPG].

Having excluded intracellular Ca^2+^ stores as the source of the α-synuclein-induced Ca^2+^ signal, we assessed whether α-synuclein species induced Ca^2+^ influx into cells from the extracellular medium. We employed the manganese (Mn^2+^)-induced quenching technique whereby fura-indicators irreversibly bind Mn^2+^ and this quenches their fluorescence. The Mn^2+^ quench process can be quantified by measuring the fluorescence of fura-2 at its isobestic wavelength of ∼360 nm. Mn^2+^, when applied extracellularly, will enter cells through Ca^2+^ permeable channels, and thus the Mn^2+^ entry through Ca^2+^ channels can be quantified using the emission of fura-2 at 360 nM (while the Ca^2+^-dependent changes of the 340 nm to 380 nm ratio of fura-2 are not altered). As a control, in the presence of monomeric (Fig. 3C; *n*=62 cells) α-synuclein but in the absence of Mn^2+^, the fura-2 signal excited at 360 nm was unaltered showing that this method reliably reports a Ca^2+^-independent signal (Fig. 3C, left *y*-axis, black). However, in the presence of 40 µM Mn^2+^ and monomeric α-synuclein (Fig. 3A) or oligomeric α-synuclein (Fig. 3B; *n*=81 cells), the fura-2 signal at 360 nm showed step-like irreversible decreases (quench) confirming the entry of Mn^2+^ through Ca^2+^ permeant channels. Each quench directly corresponds to the α-synuclein-induced transient increase in [Ca^2+^]_c_ (example traces are shown in Fig. 3A,B, right *y*-axis, red). These results suggest that the α-synuclein-induced transient [Ca^2+^]_c_ signals are caused by pulses of Ca^2+^ influx from the extracellular medium into astrocytes and neurons across the plasmalemmal membrane through Ca^2+^-permeant channels.

We then investigated whether the α-synuclein Ca^2+^ influx across the plasmalemmal membrane occurred through the known voltage-dependent Ca^2+^ channels or glutamate receptor mechanisms. We first incubated primary cultures with a variety of inhibitors of channels and receptors for 10 min, and then applied α-synuclein in order to determine their effect on the Ca^2+^ signal induced by monomers and oligomers. The concentration of these inhibitors has been previously tested and confirmed to induce inhibition of their channels or receptors. Nifedipine (1 µM) or Verapamil (20 µM), inhibitors of L-type VDCCs, did not alter the shape or amplitude of the monomeric (data not shown) or oligomeric (Fig. 3C) α-synuclein-induced [Ca^2+^]_c_ signals in either astrocytes (*n*=58 for monomers, *n*=48 for oligomers) or neurons (*n*=39, *n*=62 for both types of α-synuclein). Inhibitors of either ionotropic or metabotropic glutamate receptors, including 20 µM CNQX (*n*=156 cells), 10 µM MK-801 (*n*=286 cells) or 50 µM (S)-MCPG (*n*=215 cells), did not have any effect on the α-synuclein-induced Ca^2+^ signals, as shown on Fig. 3C. These results suggest that the monomeric- and oligomeric-induced Ca^2+^ signals are not reflective of glutamate release in the culture, and are not dependent on known voltage-dependent Ca^2+^ channels.

### Effect of monomeric α-synuclein on Ca^2+^ efflux

**Fig. 4.**
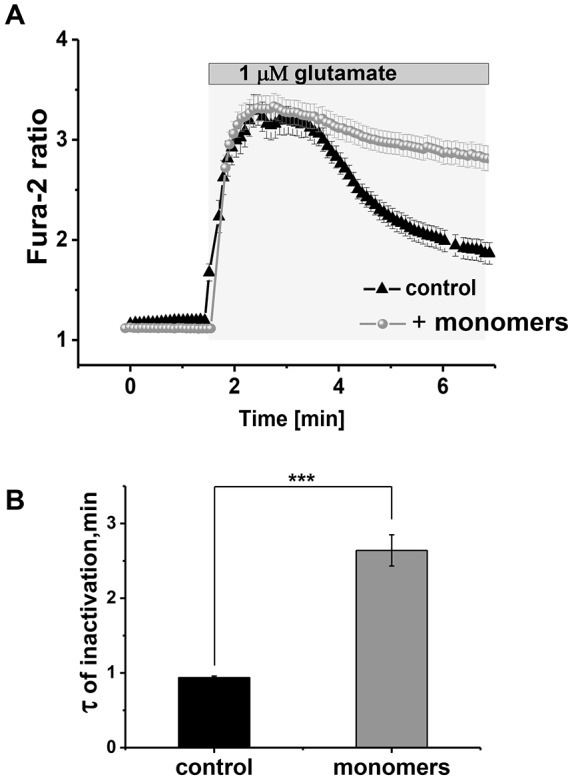
**Monomeric α-synuclein leads to impairment of Ca^2+^ efflux.** Application of 5 μM glutamate to cells pre-incubated with α-synuclein monomers (A) resulted in significantly delayed recovery of the cytosolic Ca^2+^ signal (over a period of 10 min) compared to control (recovery within 2 min, B). Results are mean±s.e.m. [*n*=91, control; *n*=68, monomers (B)]

In addition to the α-synuclein-induced Ca^2+^ signal caused by Ca^2+^ influx, we investigated whether the presence of α-synuclein was able to affect Ca^2+^ responses to physiological stimuli, such as depolarising stimuli: glutamate and KCl in neurons, and non-depolarising stimuli such as ATP in astrocytes. We therefore pre-incubated astrocytes and neurons with monomeric α-synuclein for 10 min. Subsequent stimulation of neurons with 5 µM glutamate resulted in a significantly slower [Ca^2+^]_c_ efflux in neurons when compared to control (0.936±0.021 min, *n*=91 versus 2.639±0.208 min, *n*=68, *P*<0.05; mean±s.e.m.; Fig. 4A,B).

These results suggest that monomeric α-synuclein not only induces Ca^2+^ influx into cells but also significantly inhibits Ca^2+^ efflux. This inhibition of the Ca^2+^ efflux causes the slow decay of the Ca^2+^ signal induced by stimuli and further might contribute to the slow and progressive basal [Ca^2+^]_c_ increase in neurons and astrocytes exposed to α-synuclein.

### Effect of α-synuclein on conductance of artificial lipid membranes

**Fig. 5.**
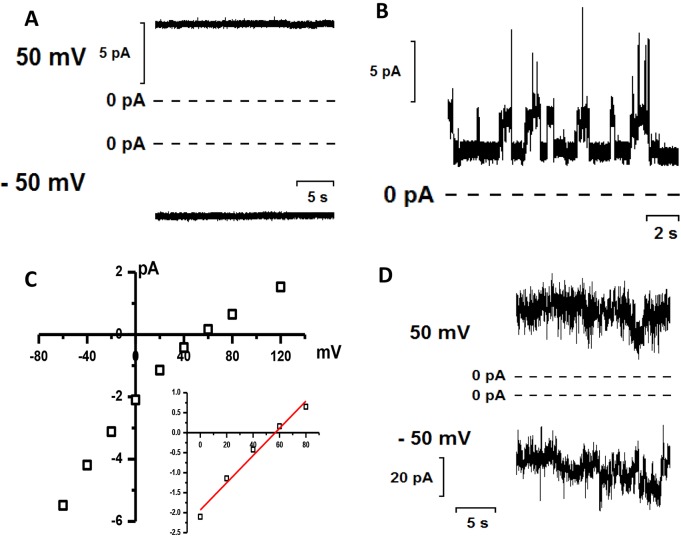
**Channel activity of α-synuclein on artificial membranes.** (A) Oligomeric α-synuclein was added to the cis compartment of a bilayer cuvette to a final concentration of 100 nM. The membrane was suspended between symmetric HBSS supplemented with 1 mM CaCl_2_. (B) Channels induced by 100 nM oligomer α-synuclein in lipid bilayers suspended between aqueous solution of 15 mM NaCl (cis) and 150 mM NaCl (trans), 2 mM CaCl_2_, 10 mM Tris-HCl pH 7.4 (symmetric). Voltage of 150 mV was applied across BLM. (C) Current–voltage dependence of the channels presented in B. (D) Irregular currents induced by 100 nM of monomeric α-synuclein in BLMs. The membrane was suspended between symmetric HBSS supplemented with 1 mM CaCl_2_.

We sought to determine the ability of α-synuclein to directly induce ionic permeability of lipid membranes and so we performed a series of experiments using phospholipid bilayer membranes and synthetic monomeric and oligomeric α-synuclein generated by aggregating fluorescently labelled α-synuclein (method 1). In our experiments we used the planar lipid bilayer (BLM) approach. This approach allows use of artificial membranes formed by pure lipids without participation of protein components, which are normally present in biological membranes. We detected that addition of oligomeric α-synuclein to the recording causes an increase in membrane permeability. We observed two distinct conductance modes consistent with channel-like behaviour of this protein. The first mode is characterized by a stable open state with no transitions to closed or sub-conductance states (Fig. 5A, conductance=92.7±2.3 pS). This type of conductance was observed in three independent experiments. The second mode is characterized by stable channel activity with distinct transitions between open and closed states (Fig. 5B, conductance 24.2±2.7 pS). It should be noted that channel activity was a relatively rare event which was detected in only ∼20% of the experiments (*n*=30). Both of these channel modes were cation selective. In the presence of a 10:1 NaCl concentration gradient across the lipid membrane, the reversal potential (zero current potential) was 57 mV, which is close to the theoretical Nernst potential for Na^+^ selectivity under these conditions (58 mV) (Fig. 5C). These channels, however, were not selective between Na^+^ and Ca^2+^. Importantly, channel activity was detected only in the presence of oligomeric, but not monomeric, α-synuclein species. We should note that in addition to the channel activity, we detected irregular ionic currents in the bilayer (Fig. 5D). This activity was observed in the presence of monomeric as well as in the presence of oligomeric samples. This suggests that the irregular conductance in the presence of oligomeric sample was likely due to the presence of monomeric α-synuclein in the sample. These data show that both monomeric and oligomeric α-synucleins are capable of increasing the permeability of lipid membranes. However, only oligomeric forms can form ion channels with stable conductance.

Overall these data suggest that oligomers might disrupt lipid membranes through different mechanisms that might depend on the lipid composition, the curvature of the lipid bilayer and the protein-to-lipid ratio.

### Ca^2+^ signalling and toxicity

**Fig. 6.**
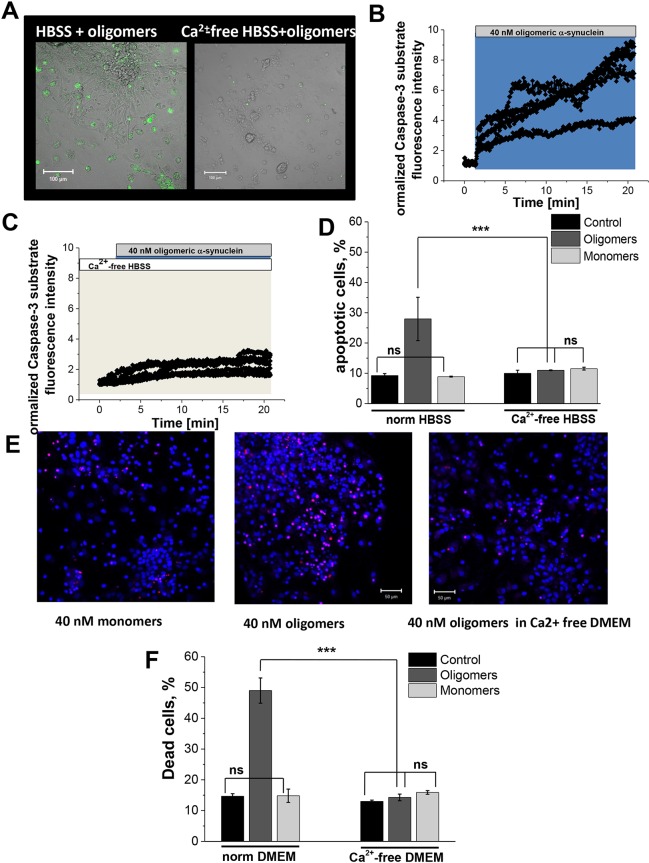
**Protective effect of Ca^2+^-free medium against α-synuclein-induced caspase-3 activation and cell death.** (A,B,D) 40 nM oligomeric α-synuclein significantly activates the NucView 488 caspase 3 substrate in neurons and astrocytes. (C) Pre-incubation in Ca^2+^-free medium significantly reduced caspase 3 activation, shown as the time from addition of the peptide to activation of the substrate (i.e. increase of fluorescence in A). (D) Quantification of α-synuclein-induced apoptosis in Ca^2+^-containing and Ca^2+^-free medium. (E) Cell death was assessed using propidium iodide to label dead cells and Hoechst 33342 to label all cells. (F) Quantification of α-synuclein-induced cell death in normal and Ca^2+^-free medium. Results are mean±s.e.m. [*n*=69 (D); *n*=32 (norm); *n*=37 (Ca^2+^-free) (F)]. ***P<0.001; ns, not significant (Student's *t*-test).

Given that Ca^2+^ dysregulation is known to be a key player in cell death induction, we investigated whether the α-synuclein-induced Ca^2+^ signal leads to neurotoxicity and ultimately cell death. We treated primary co-cultures with monomeric or oligomeric α-synuclein, assessed caspase-3 activation using the fluorescent-labelled caspase-3 substrate NucView and recorded the number of cells undergoing apoptosis over a 30-min period. We found that within 15 min, a significant percentage of cells exposed to oligomeric, but not monomeric α-synuclein, started to undergo apoptosis (representative images shown in Fig. 6A, representative traces of cells loaded with NucView shown in Fig. 6C). We then applied the same monomeric and oligomeric α-synuclein preparations to cells in Ca^2+^-free HBSS. The percentage of cells undergoing apoptosis induced by oligomeric α-synuclein was reduced significantly (from 27.9±7.2, *n*=32 to 11.5±0.5, *n*=37, *P*=0.0012; Fig. 6B). In order to assess prolonged exposure to α-synuclein, we incubated the cells in normal and Ca^2+^-free Dulbecco's modified Eagle medium (DMEM), containing monomeric or oligomeric α-synuclein for 6 h, and counted the number of Hoechst-33342- or propidium-iodide-positive cells (representative images shown in Fig. 6D). We showed that oligomeric α-synuclein, but not monomeric α-synuclein, induced significant cell death after 6 h, which was prevented by exclusion of Ca^2+^ from the extracellular medium (from 49.0%±4.1, *n*=32 to 14.3%±1.1, *n*=37, *P*=0.0302; Fig. 6E).

## DISCUSSION

In this study, we sought to unravel the link between misfolded α-synuclein protein and aberrant Ca^2+^ signals, and dyshomeostasis and neuronal death in Parkinson's disease. Extracellular α-synuclein is taken up by cells rapidly and is responsible for subsequent transmission of pathology, both in terms of inducing neuronal death as well as seeding further aggregation. However, the initial pathophysiological effect of extracellular α-synuclein on a cell is not well delineated, and the species-specific form of extracellular α-synuclein and its correlative cellular effects are also poorly established. Our model of applying different conformational species of α-synuclein to cells and observing the immediate effects on Ca^2+^ signalling is therefore highly relevant to understand the species-specific effects of α-synuclein. We utilised recombinant α-synuclein at different stages of aggregation and applied these preparations to neurons and astrocytes. Detailed biophysical characterisation of two different preparations of oligomers confirm that we have generated oligomers that are stable, resistant to proteinase K degradation and rich in β-sheet structure. Such oligomers are formed late in the aggregation process and are proposed to be the more toxic to cells owing to their ability to interact with membranes. We showed that exogenously applied monomers and oligomers are in fact both able to induce cytosolic Ca^2+^ signal at nanomolar concentrations in a range of models: primary neurons, iPSC-derived neurons and acute brain slices. Extracellular α-synuclein was able to induce two different patterns of cytosolic Ca^2+^ signalling: (1) a gradual increase in the basal cytosolic Ca^2+^ level and (2) transient Ca^2+^ spikes. Patterns 1 and 2 could occur distinctly or in combination in the same cell. However, the monomer- and oligomer-induced Ca^2+^ signal was entirely dependent on the presence of extracellular Ca^2+^. Thus, both monomeric and oligomeric α-synuclein induce a cytosolic Ca^2+^ signal in cells by inducing pulses of Ca^2+^ influx across the plasmalemmal membrane through Ca^2+^-permeable channels.

It is well established that α-synuclein binds negatively charged phospholipids, and thus binds to membranes. Monomeric α-synuclein assumes α-helical structure on binding which is thermodynamically favourable, whereas the oligomeric (or protofibrillar form) binds membranes with higher affinity than monomers and retains a β-sheet structure. There are currently two major biophysical hypotheses that explain the Ca^2+^ influx seen with aggregated α-synuclein. First is the amyloid pore hypothesis in which oligomeric α-synuclein is able to form pore-like structures with non-selective leakage of compounds (Volles et al., 2001). Atomic force microscopy, circular dichroism and electrophysiology have demonstrated the formation of ion channels by soluble amyloid subunits in membrane bilayers that display multilevel conductances reflective of the number of units forming the channel (Quist et al., 2005). The second hypothesis is that the incorporation of oligomeric species in between tightly packed lipids in the bilayer membrane results in reduced lipid order, thinning of the membrane and reduction in the dielectric barrier and an increase in membrane permeability (and hence conductance) in the absence of ion channel formation (Kayed et al., 2004; Sokolov et al., 2006).

Monomers of α-synuclein have also been reported to affect membranes through differing mechanisms, that is, altering permeability or inducing channel formation. High concentrations of monomers have been shown to permeabilise membranes through detergent-like mechanisms (Volles and Lansbury, 2002). One study has demonstrated that monomeric α-synuclein formed discrete highly conductive ion channels in planar BLMs in the presence of a transmembrane potential. Importantly, these channels are voltage sensing as the structural changes in α-synuclein were dependent on the membrane potential, favouring insertion into depolarised membranes (Zakharov et al., 2007).

In order to understand the effect of our monomeric and oligomeric species of α-synuclein on membranes, we applied them to artificial bilayer membranes and recorded changes in conductance. In agreement with the monomeric and oligomeric α-synuclein-induced Ca^2+^ signal in cells, our bilayer membrane data suggest that the presence of both monomeric and oligomeric α-synuclein causes an increase in lipid membrane permeability. In the presence of monomeric α-synuclein, membrane conductance was highly irregular with frequent ‘spikes’ of ionic currents. Conversely, in the presence of oligomeric α-synuclein, we detected ion currents with distinct stable conductance levels, which are typical for ion channels and pores. For both monomers and oligomers, their effect on cell membrane conductance and Ca^2+^ influx can therefore be explained by their direct interaction between cell lipid membranes and the protein. Overall, our data is in agreement with both hypotheses of the interaction of monomers and oligomers with membranes: lipid thinning and resultant increase in permeability leads to ionic currents and Ca^2+^ spiking with both monomers and oligomers. However, in the case of oligomers, membrane permeabilisation produces a stronger effect likely due to the formation of stable pores resulting in rare, but regularly occurring, ion channel formation. Thus the α-synuclein-induced Ca^2+^ signal seen in cells is due to the interaction of monomeric and oligomeric α-synuclein with the neuronal membrane affecting its permeability and allowing different types of Ca^2+^ influx to occur.

Finally, we wished to understand the relationship between the aberrant Ca^2+^ signals, impaired Ca^2+^ homeostasis and cell death in Parkinson's disease. We report that although monomeric and oligomeric forms of α-synuclein both induce Ca^2+^ signals, it is only the oligomeric species that induces apoptosis and cell death when applied to cells. Furthermore, the toxicity of the oligomeric α-synuclein can be abolished by preventing the Ca^2+^ influx through removal of extracellular Ca^2+^. This finding supports the fact that β-sheet-rich oligomeric species are the most toxic species in cells, and that this toxicity is crucially dependent on Ca^2+^ dysregulation. We have previously shown that the β-sheet-rich oligomeric species (and not the monomeric species) generates reactive oxygen species (ROS) resulting in oxidative stress (Cremades et al., 2012; Deas et al., 2015), and also that oligomeric species (and not monomeric species) can induce lipid peroxidation and cell death (Angelova et al., 2015). Therefore, we hypothesise that the Ca^2+^ dysregulation together with the increased levels of ROS induced by α-synuclein oligomers act synergistically to result in cell toxicity. The monomeric-induced Ca^2+^ dysregulation is insufficient (without a concomitant increase in ROS) to induce cell death in this timeframe. Interestingly it has been reported that α-synuclein cytotoxicity in a yeast model is mediated by the ability of α-synuclein to increase the basal intracellular Ca^2+^ levels (through a PMR1-mediated mechanism) and this results in an oxidative burst that ultimately kills cells. Abolishing the raised intracellular Ca^2+^ prevents cell death in this model (Buttner et al., 2013). This model of α-synuclein strongly resembles the effects of β-amyloid, which also is able to induce permeability of membranes and a Ca^2+^ signal, which together with ROS, can initiate cell death (Abramov et al., 2003).

In conclusion, the combined data from Ca^2+^ imaging in primary neurons exposed to α-synuclein and bilayer membrane experiments point us towards a unifying mechanism: increased levels of endogenous or exogenous α-synuclein in its monomeric form can alter lipid permeability and induce Ca^2+^ spiking and changes in basal Ca^2+^ due to impaired efflux. During the aggregation process, conversion of the monomeric α-synuclein into oligomeric α-synuclein that has a β-sheet structure results in insertion of oligomeric α-synuclein into membranes inducing ion channel formation and further Ca^2+^ influx. Initially this might lead to dysfunction of Ca^2+^-dependent mechanisms such as synaptic neurotransmission and plasticity. However, ultimately under conditions of altered clearance and buffering of Ca^2+^, the Ca^2+^ dysregulation is sufficient to result in Ca^2+^-dependent cell death and neurodegeneration.

## MATERIALS AND METHODS

### Generation and characterisation of monomeric and oligomeric α-synuclein

The A90C mutant variant of α-synuclein was expressed in, and purified from, *E. coli* as described previously (Hoyer et al., 2002), and labelled with either maleimide-modified AF488 or AF647 dyes (Invitrogen), through the cysteine thiol moiety (at labelling efficiencies of 98±3% for both cases as estimated by mass spectrometry). The labelled protein was purified from the excess of free dye by a P10 desalting column with Sephadex G25 matrix (GE Healthcare), divided into aliquots, flash frozen and lyophilised; the lyophilised protein was stored at −20°C. Fluorescently-labelled oligomers (method 1) were generated by using 1 mg/ml wild-type (WT) α-synuclein, AF647-labelled protein or equimolecular concentrations of AF488- and AF647-labelled protein (1 mg/ml total protein concentration) in 25 mM Tris-HCl pH 7.4, 0.1 M NaCl with 0.01% NaN_3_ to avoid bacterial growth during sample incubation. The samples were incubated in Eppendorf tubes at 37°C under constant shaking at 200 rpm. For the single-molecule FRET analysis of the fluorescently labelled oligomers, a 2-µl aliquot was diluted 10^5^-fold by serial dilution with 0.022-µm-filtered 25 mM Tris-HCl pH 7.4, 0.1 M NaCl. Glass slides were incubated for 1 h with bovine serum albumin (BSA) at 1 mg/ml to prevent α-synuclein species from adsorbing to the surface, as we have recently shown (Cremades et al., 2012). Immediately after removal of the BSA solution, 500 µl of diluted sample was placed on the slide for analysis.

For, method 2, human α-synuclein was overexpressed and purified as a monomeric fraction from *E. coli* as described previously (Hoyer et al., 2002). Samples of α-synuclein oligomers were prepared by incubating monomeric protein at ∼800 µM (12 mg/ml) in PBS at 37°C without agitation for 18–22 h. To get this high concentration of protein, a solution of monomeric α-synuclein in milliQ water was lyophilised and, typically, 6 mg of lyophilised α-synuclein was resuspended in 500 µl of PBS buffer. The resulting solution was passed through a filter device with a 0.22-µm cut-off to remove any particles of dust and/or large protein aggregates that could have been formed during the lyophilisation process. After incubating this solution at 37°C, without agitation, for 18–22 h, most of the protein remains monomeric, but a fraction of ∼5% of the protein is in the form of aggregates. These aggregates are still soluble after ultracentrifugation at 90,000 rpm for 1 h. In order to isolate these aggregates from the monomeric protein, a series of filtering devices with a 100-kDa cut-off were used, so that the monomeric protein passed through the membrane while oligomeric species bigger than 100 kDa remained at the top of the filter.

### Characterisation of α-synuclein in planar lipid bilayers

Phospholipids 1,2-dioleoyl-*sn*-glycero-3-phosphocholine (DOPC) and cardiolipin were obtained from Avanti Polar Lipids (Alabaster, AL). Planar BLMs were formed from a 20 mg/ml lipid solution of either DOPC or DOPC:Cardiolipin (3:1) in n-decane (Aldrich) as has been shown previously (Abramov et al., 2011). The solution was painted across the 200-µm aperture of a Delrin cup (Warner Instruments, Hamden, CT). Both cis (voltage command side) and trans (virtual ground) compartments of the cuvette contained HBSS solution containing 5.3 mM KCl, 138 mM NaCl, 1 mM CaCl_2_, 0.4 mM KH_2_PO_4_, 0.3 mM Na_2_HPO_4_, 4.2 mM NaHCO_3_. 100 nM of α-synuclein was added to the cis compartment of the cuvette. All measurements were performed at room temperature.

For the measurements of the selectivity of the induced conductance, reversal potential (zero current potential) was measured in a presence of 10:1 (trans-to-cis) transmembrane ion gradient. In these experiments, membranes were formed between compartments containing: (cis) 15 mM NaCl, 2 mM CaCl_2_, 10 mM Tris-HCl pH 7.4 and (trans) 150 mM NaCl, 2 mM CaCl_2_, 10 mM Tris-HCl pH 7.4. The liquid junction potential offset was compensated for prior to the membrane formation.

Currents across the bilayer were recorded using a Planar Lipid Bilayer Workstation (Warner Instruments). The cis compartment was connected to the head stage input and the trans compartment was held at virtual ground by a pair of matched Ag–AgCl electrodes. Signals from voltage-clamped BLMs were high-pass-filtered at 2.1 kHz using an eight-pole Bessel filter LPF-8 (Warner Instruments), digitized using Data Translation Digitizer and recorded on a computer using software developed by Elena Pavlova (available on request).

### Cell culture

Mixed cultures of hippocampal or cortical neurons and glial cells were prepared as described previously (Vaarmann et al., 2010) with modifications, from Sprague-Dawley rat pups 2–4 days post-partum (UCL breeding colony). Experimental procedures were performed in full compliance with the United Kingdom Animal (Scientific Procedures) Act of 1986. Hippocampi and cortex were removed into ice-cold PBS (Ca^2+^- and Mg^2+^-free, Invitrogen, Paisley, UK). The tissue was minced and trypsinised (0.25% for 5 min at 37°C), triturated and plated on poly-D-lysine-coated coverslips and cultured in Neurobasal A medium (Invitrogen, Paisley, UK) supplemented with B-27 (Invitrogen, Paisley, UK) and 2 mM L-glutamine. Cultures were maintained at 37°C in a humidified atmosphere of 5% CO_2_ and 95% air, fed twice a week and maintained for a minimum of 12 days before experimental use to ensure expression of glutamate and other receptors. Neurons were easily distinguishable from glia: they appeared phase bright, had smooth rounded somata and distinct processes, and lay just above the focal plane of the glial layer. Cells were used at 12–15 days *in vitro* (DIV).

### Acute brain slices

Horizontal hippocampus or entorhinal cortex slices (100 µm) were prepared from P9 Wistar rats, using standard procedures. Tissue was sliced at ∼4°C in artificial cerebrospinal fluid (in mM: NaCl, 129; NaH_2_PO_4_, 1.25; glucose, 10; MgSO_4_, 1.8; CaCl_2_, 1.6; KCl, 3; NaHCO_3_, 26, pH 7.4) continuously bubbled with 95% O_2_ and 5% CO_2_. Slices were imaged in HBSS for up to 3 h after preparation.

### Imaging [Ca^2+^]_c_

Primary neurons and astrocytes were loaded for 30 min at room temperature with 5 µM fura-2 AM or fluo-4 AM and 0.005% pluronic in a HEPES-buffered salt solution (HBSS) (in mM: 156 NaCl, 3 KCl, 2 MgSO_4_, 1.25 KH_2_PO_4_, 2 CaCl_2_, 10 glucose and 10 HEPES, pH adjusted to 7.35 with NaOH). Ca^2+^-free medium contained 0.5 mM EGTA.

Fluorescence measurements were obtained on an epifluorescence inverted microscope equipped with a 20× fluorite objective. [Ca^2+^]_c_ was monitored in single cells using excitation light provided by a xenon arc lamp, the beam passing monochromator at 340, 360 and 380 nm (Cairn Research, Kent, UK). Emitted fluorescence light was reflected through a 515-nm long-pass filter to a cooled CCD camera (Retiga, QImaging, Canada) and digitised to a 12-bit resolution. All imaging data were collected and analysed using software from Andor (Belfast, UK). Fura-2 is a ratiometric dye with a high affinity for Ca^2+^ and this allows an accurate measurement of the cytosolic Ca^2+^ independent of loading variations. The experimental setup used for measuring fura-2 also enables signals with transient rapid kinetics and decay times to be measured. The measurement of this dye is a ratio between the emitted light at 340 nm (high Ca^2+^) and the emitted light at 380 nm (low Ca^2+^). The fura-2 data have not been calibrated in terms of [Ca^2+^]_c_ because of the uncertainty arising from the use of different calibration techniques.

Confocal images were obtained using a Zeiss 710 CLSM equipped with a 40× oil immersion objective. The 488-nm argon laser line was used to excite fluo-4 which was measured between 510–550 nm. Illumination intensity was kept to a minimum (at 0.1–0.2% of laser output) to avoid phototoxicity and the pinhole set to give an optical slice of ∼2 µm. All data presented were obtained from at least five coverslips and two or three different cell preparations. Fluo-4, in contrast is a single-wave Ca^2+^ sensor where the emission intensity depends on the levels of bound Ca^2+^. It has a lower *K*_d_ and is useful for visualising Ca^2+^ variations around the basal concentration. As fluo-4 intensity is measured on the confocal microscope, the units used for these experiments are (arbitrary) fluorescence units.

### Toxicity experiments

For toxicity assays, we loaded cells simultaneously with 20 µM propidium iodide, which is excluded from viable cells but exhibits a red fluorescence following a loss of membrane integrity, and 4.5 µM Hoechst 33342 (Molecular Probes, Eugene, OR), which gives a blue staining to chromatin, to count the total number of cells. Using phase-contrast optics, a bright-field image allowed identification of neurons, which look quite different to the flatter glial component and also lie in a different focal plane, above the glial layer. A total number of 600–800 neurons or glial cells were counted in 10 fields of each coverslip. Each experiment was repeated five or more times using separate cultures.

### Caspase-3 activity assay

For measurements of caspase-3 activation, cells were loaded for 15 min at room temperature with 10 μM NucView 488 caspase-3 substrate (Biotium, USA) in HBSS. NucView 488 is a novel class of enzyme substrates for real-time detection of caspase-3 activity in live cells. The substrate can rapidly cross the cell membrane to enter the cell cytoplasm, where it is cleaved by caspase-3 to release the high-affinity DNA dye. The released DNA dye migrates to the cell nucleus to stain the nucleus brightly green.

Confocal images were obtained using Zeiss 710 confocal laser scanning microscope and a 40× oil immersion objective. The 488-nm argon laser was used to excite NucView 488 fluorescence, which was measured using a bandpass filter from 510 and 560 nm.

### Statistical analysis

Statistical analysis and exponential curve fitting were performed using Origin 9 (Microcal Software Inc., Northampton, MA) software. Results are expressed as mean±s.e.m. Student's *t*-tests were performed where appropriate and *P*-values provided in results section.
